# Correlation between the Altered Gut Microbiome and Lifestyle Interventions in Chronic Widespread Pain Patients: A Systematic Review

**DOI:** 10.3390/medicina59020256

**Published:** 2023-01-29

**Authors:** María Elena Gonzalez-Alvarez, Eleuterio A. Sanchez-Romero, Silvia Turroni, Josué Fernandez-Carnero, Jorge H. Villafañe

**Affiliations:** 1 Department of Physical Therapy, Occupational Therapy, Rehabilitation and Physical Medicine, Rey Juan Carlos University, 28032 Madrid, Spain; 2 Escuela Internacional de Doctorado, Rey Juan Carlos University, 28008 Madrid, Spain; 3 Department of Physiotherapy, Faculty of Sport Sciences, Universidad Europea de Madrid, 28670 Villaviciosa de Odón, Spain; 4 Physiotherapy and Orofacial Pain Working Group, Sociedad Española de Disfunción Craneomandibular y Dolor Orofacial (SEDCYDO), 28009 Madrid, Spain; 5 Musculoskeletal Pain and Motor Control Research Group, Faculty of Sport Sciences, Universidad Europea de Madrid, 28670 Villaviciosa de Odón, Spain; 6 Department of Physiotherapy, Faculty of Health Sciences, Universidad Europea de Canarias, 38300 Santa Cruz de Tenerife, Spain; 7 Musculoskeletal Pain and Motor Control Research Group, Faculty of Health Sciences, Universidad Europea de Canarias, 38300 Santa Cruz de Tenerife, Spain; 8 Department of Pharmacy and Biotechnology, University of Bologna, Via Belmeloro 6, 40126 Bologna, Italy; 9 IRCCS Fondazione Don Carlo Gnocchi, 20148 Milan, Italy

**Keywords:** gut microbiome, lifestyle, chronic widespread pain

## Abstract

*Background*: Lifestyle interventions have a direct impact on the gut microbiome, changing its composition and functioning. This opens an innovative way for new therapeutic opportunities for chronic widespread patients. *Purpose*: The goal of the present study was to evaluate a correlation between lifestyle interventions and the gut microbiome in patients with chronic widespread pain (CWP). *Methods*: The systematic review was conducted until January 2023. Pain and microbiome were the two keywords selected for this revision. The search was conducted in PubMed, Chochrane, PEDro and ScienceDirect, where 3917 papers were obtained. Clinical trials with lifestyle intervention in CWP patients were selected. Furthermore, these papers had to be related with the gut microbiome, excluding articles related to other types of microbiomes. *Results*: Only six articles were selected under the eligibility criteria. Lifestyle interventions were exercise, electroacupuncture and ingesting a probiotic. *Conclusions*: Lifestyle intervention could be a suitable choice to improve the gut microbiome. This fact could be extrapolated into a better quality of life and lesser levels of pain.

## 1. Introduction

One in three people experience chronic pain, and at least one in ten experience an even greater symptomatology called chronic widespread pain (CWP), which carries a cost in absenteeism from work and represents a burden on the national health system and a worldwide problem [1,2,3]. CWP is a condition of diffuse musculoskeletal pain associated with other illness, which presented axial pain on both sides of the body [4]. The latest scientific literature has described the mechanisms underlying the communication between the gut and the brain (i.e., the gut–brain axis [5]), which could partly explain the chronicity of pain, as well as pave the way for new therapeutic opportunities for this population [2,6]. In particular, it is known that abnormalities in the gut microbiome (i.e., dysbiosis) could lead to systemic inflammation, especially in the presence of an impaired intestinal barrier (the so-called leaky gut) [7,8]. Moreover, some intestinal microorganisms can influence the production of neurotransmitters (and produce them by themselves) in addition to directly stimulating nerve fibers, also interfering in the hypothalamus–pituitary–adrenal axis [9]. Alterations along the gut–brain axis have been shown to be related with musculoskeletal pain, behavior modulation or brain processing, and play a role in depression, stress, anxiety and even neuropsychiatric disorders [10,11,12,13].

Lifestyle interventions such as nutrition, sleep or exercise could affect the pain experience [14,15,16]. Specifically, physical activity has a direct impact on the central nervous system (CNS), modifying the pain experience and cognitive processing [14,17]. On the other hand, it has been shown to positively modulate the gut microbiome (leading to greater diversity and overabundance of beneficial taxa and metabolites) in different settings, including chronic diseases [17,18] thus, representing a potential preventive and therapeutic tool for dysbiosis-related conditions.

As far as we are aware, the bibliography related to these three items (pain, microbiome and lifestyle) is still limited. Despite there being a wide bibliography about the effects of exercise in CWP patients, there is only one article in which exercise is the chosen intervention regardless of microbiota changes [18]. This stresses the need for further research in this very promising field, which could open a new vision and indicate new therapeutic targets for CWP patients.

Here, we provide an up-to-date systematic review of studies investigating the correlation between the gut microbiome and lifestyle interventions in CWP. In particular, the associations between gut microbiome and pain, quality of life and exercise are discussed.

## 2. Materials and Methods

### 2.1. Data Source and Search Strategy

A systematic review was conducted until January 2023 that investigated the correlation between the gut microbiome and lifestyle interventions in CWP. Preferred Reporting Items for Systematic Reviews and Meta-Analyses (PRISMA) guidelines were followed.

The criteria used to extract the data were according to the *Cochrane Handbook for Systematic Reviews of Interventions*(Version 6.3, 2022) [19]. The data selected were study design, age of participants, year and country of publication, setting, intervention, follow-up timing, clinical outcomes and reported findings. The protocol has been registered on 18 November 2022 in the International Prospective Register of Systematic Reviews (PROSPERO, CRD42022373890).

The principal researcher conducted the systematic review according to the PRISMA criteria by inserting the keywords “pain” and “microbiome”, combined with the Boolean “AND” in PubMed, Cochrane, PEDro and ScienceDirect. This strategy was reviewed by two other authors. The whole search strategy used was: (“pain”[MeSH Terms] OR “pain”[All Fields]) AND (“microbiome”[All Fields] OR “microbiomic”[All Fields] OR “microbiomics”[All Fields] OR “microbiota”[MeSH Terms] OR “microbiota"[All Fields] OR “microbiome”[All Fields] OR “microbiomes”[All Fields]).

### 2.2. Eligibility Criteria

Eligibility criteria were: (1) clinical trial, (2) lifestyle intervention, (3) patients with CWP (4) in the gut microbiome and (5) until January 2023.

### 2.3. Data Extraction

Relevant articles were identified by the principal investigator (MEGA) and reviewed by two other authors (EASR and JHV). Discrepancies were resolved by the consensus of the three researchers. Researchers were not blind to any information regarding the authors, the journal or the outcomes for each article reviewed. The data extracted from the studies included study design, participants, intervention, outcome measures, follow-up and reported results (Table 1).

### 2.4. Outcome Measures

The primary outcome was the change in pain between baseline and follow-up. Furthermore, quality of life, scales related to their CWP symptoms and exercise were also added as secondary measures.

### 2.5. Quality Assessment

All articles selected by the principal author (MEGA) were assessed by two different authors (EASR and JHV), using two different scales for methodological quality. Control trial studies were evaluated with the Physiotherapy Evidence Database (PEDro) Scale (Table 2), while longitudinal studies were evaluated using the Methodological Index for Nonrandomized Studies (MINORS) (Table 3). Disagreements were solved by the three authors cited above. The entire quality assessment process was developed based on previous studies [20,21,22,23].

## 3. Results

### 3.1. Study Selection

A total of 3917 studies were identified by searching the PubMed, Cochrane, PEDro and ScienceDirect databases. No other studies were added from other sources. After removing the duplicates and screening titles, abstracts, and full texts, we selected eight articles. In a secondary screening, one from PubMed was discarded because the intervention was not completely related with our topic, and one from Cochrane was discarded because the registration of the Roman et al. [12] manuscript was included in the PubMed section. Six articles were therefore selected for this review, with 337 participants in total. Three studies were conducted in Europe (Spain, Italy and Denmark) and three in Asia (China and Turkey).

**Figure 1:**
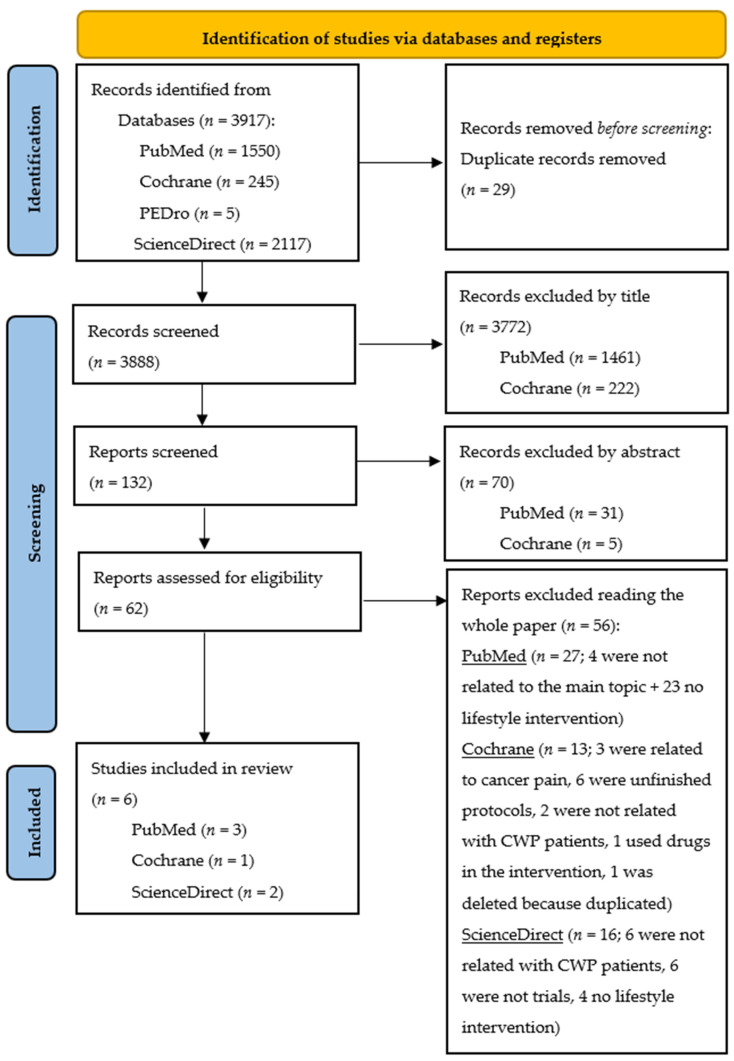
PRISMA 2020 flow diagram for new systematic reviews, including searches of databases and registers.

Although Roman et al. [12] and Jensen et al. [24] did not profile the gut microbiome, their works were included as they used probiotics and well-known microbiome manipulation tools [25]. The same scenery can be found in the Kenis-Coskun et al. [4] manuscript but in this case with Vitamin D [4]. Otherwise, Torlak et al. manipulated the diet and used different diets with chronic low back pain (CLBP) patients [26]. The other two studies investigated the effect of a maximal exercise challenge in myalgic encephalomyelitis/chronic fatigue syndrome (ME/CFS) [18] and the effect of electroacupuncture in knee osteoarthritis patients [27]. The flowchart is presented in Figure 1 according to the PRISMA guidelines.

**Table 1:** Studies data extraction.

Author, Year.	Study Design	Participants	Intervention	Outcome Measures	Reported Results
Shukla et al., 2015 [18]	Randomized controlled trial	20 subjects (10 ME/CFS patients and 10 controls) Mean age of patients was 48.6 years Mean age of controls is 46.5 years Inclusion criteria: subjects with CFS without any other major illness	Maximal exercise test on an electronically braked cycle ergometerDuration of trial: 3-min warm-up at 25 W and the rate was increased to 5 W every 20 s. Participants should maintain a pedal rate between 60–70 RPM. The test ended if the participant could not maintain the pedal rate or stopped pedaling	Sample collection (blood and stool) DNA sequence analysis MFI POMS McGill Pain Questionnaire Symptom Inventory: Diarrhea and Stomach/Abdominal Pain Symptom Inventory: Neurocognitive Symptoms Memory ProblemsConcentration Problems Measured and Follow-up: Pre-intervention, immediately pre-test, immediately post-test, 40 h and 72 h post-test	Baseline: statistically significant differences between groups in MFI and POMS. A significant difference between groups in heart rate during the test. The relative abundance of Actinobacteria in the gut microbiome was significantly lower in ME/CFS patients than in healthy controls. Authors’ conclusions: “Exercise induced bacterial translocation, one likely argument to why patients worsen when they try to be more physically active.”
Roman et al., 2018 [12]	Randomized controlled trial	31 fibromyalgia participants Inclusion criteria: >1 year from the fibromyalgia diagnosed before the study. Exclusion criteria (1) use of antibiotics and nutritional supplements (2) allergies (3) participating in other psychological or medical studies (4) pregnant/ (5) breastfeeding (6) severe intestinal disease (7) psychiatric disorders	Probiotic ingests (*n* = 16) Ctrl: sham probiotics (*n* = 15) 2 pills before breakfast and dinner, for 8 weeks	VAS FIQ SF-36 STAI BDI MMSE Cognitive tasks (choice task and the Iowa gambling task) Cortisol Measures and Follow-up Pre- and post-intervention	Both groups: ↓ FIQ, depressive symptoms and cortisol ↑ SF-36 Authors’ conclusions: “This intervention improves cognition, specifically impulsive choice and decision-making, in a group of patients diagnosed with fibromyalgia.”
Jensen et al., 2019 [24]	Randomized double-blind placebo controlled trial 1 year follow-up	85 participants, 42 active capsules and 43 placebo capsules. Inclusion criteria: CLBP and MC1 Age 18–65 Danish speakers RMQ < 5 Exclusion criteria: Intended, planned or previous back surgery Planned or current (last 3 months) antibiotic treatments for MC, immunosuppressants, intestinal pathology, immune deficiency, cancer or inability to complete de project	Active group: Probiotic Dicoflor ^®^ twice daily for 100 days. Each capsule contains 6 billion Lactobacillus Rhamnosis GG. Placebo group: Placebo capsules indistinguishable from Dicoflor twice daily for 100 days	Age and sex Pain duration and intensity (NRS) Likert scale BMI RMQ Back+leg pain by the LBP rating scale Measures and Follow-up Until 1 year follow-up	No differences between intervention groups in regard to the predefined outcomes disability, back + leg pain, patient-reported global effect or the number of the patients with minimal disability at 1 year. Back pain decreased a little more in the active intervention group than in the control group. Authors’ conclusions: “The study confirmed that treatment with probiotics, was safe and implicated no more side effects than placebo.”
Kenis-Coskun et al., 2020 [4]	Clinical Trial	51 Female patients who have CWP with vitamin D deficiency Mean age: 44.3 ± 12.7 Mean symptom duration was 13.1 ± 6.7 months. Mean BMI: 21.6 ± 3.9 Exclusion criteria: Medications that alter CPS neuropathies Comorbidities that can cause vitamin D deficiency Rheumatologic or metabolic diseases Surgery (last 6 months) CNS disorders Psychiatric disorders BMI < 30.	8-week replacement therapy of vitamin D	VAS LANSS QoL (NHP) CSP parameters Vitamin D measurements Measures and Follow-up Before and after treatment	No significant changes un CSP parameters with Vitamin D replacement. Vitamin D replacement improve pain levels and QoL in patients with CWP. Authors’ conclusions: “These results imply that in whichever way vitamin D is effective in CWP, it does not seem to be via the spinal inhibitory circuit that elicits the inhibition of muscle contraction with painful stimuli.”
Wang et al., 2021 [27]	Randomized controlled trial	90 participants (60 knee OA patients + 30 healthy controls) Inclusion criteria: Age 45–75 years, radiographically confirmed OA on one or both knees for more than 6 months and pain intensity ≥4 out of 10. Exclusion criteria: knee surgery, floating cartilage, joint effusion, inflammatory, malignant, or autoimmune disease, serious acute or chronic organic disease or mental disorder, pregnancy or breastfeeding, or history of bleeding disorder Participants were not included if they had acupuncture treatment or participated in other clinical trials in the past 3 months.	3 groups: EA: acupoints selected by formed acupuncturists (*n* = 30) SA: sham acupoints (*n* = 30) Healthy controls (*n* = 30) Duration of trial: 8 weeks	WOMAC (pain, stiffness and function subscales) NRS Function subscale: SF-12 Physical and mental health summary Fecal sample and DNA extraction 16S ribosomal RNA gene sequencing Microbial analysis Measures and Follow-up 0, 4, 8, 16, and 26 weeks of follow-up	↑ WOMAC total scores at 8 weeks in EA compared to SA ↑ WOMAC pain scores at 8 weeks in EA compared to SA ↓ NRS scores at 8 weeks in EA compared to SA Microbiota profiles were significantly different between EA (before intervention) and healthy controls. Blautia, Streptococcus and Eubacterium ↑ and Bacteroides and Agathobacter ↓ in EA. After treatment, Agathobacter and Lachnoclostridium ↑ EA. - Bacteroides were - correlated with NRS score, WOMAC total score, and WOMAC pain, stiffness and function. - - Agathobacter was - correlated with NRS score, WOMAC total score, and WOMAC pain, stiffness and function scores. - - Faecalibacterium was - correlated with NRS score, WOMAC total score, and WOMAC pain and function. - - Roseburia was - correlated with WOMAC total score, and WOMAC pain, stiffness and function. - Streptococcus was + correlated with NRS score, WOMAC total score, and WOMAC pain, stiffness and function scores. - - Enterococcus was + correlated with NRS score and WOMAC pain score. - Eubacterium, Blautia and Anaerostipes were positively correlated with SF-12 physiological score and SF-12 psychological score Authors’ conclusions: - EA was more effective than SA at 8 weeks - EA treatment modified the diversity of the gut microbiome
Torlak et al., 2022 [26]	Randomized controlled trial	60 sedentary, CLBP patients (30 female + 30 male) Inclusion criteria: VAS > 5 BMI > 25 kg/m^2^. Exclusion criteria: Individuals who engage in active exercise Intake of painkillers, anti-depressant or cortisone Pregnant individuals; Severe chronic illness Spine surgery	PTG (*n* = 20) PT+DG (*n* = 20) DG (*n* = 20) DG lasted for 5 weeks, monitored daily PTG lasted for 5 weeks, monitored 5 times a week. Program: hot packs + US + TENS PT+DG used both intervention	BMI VAS LANSS BI Measures and Follow-up Before and after treatment	Significant difference in VAS scores in each group before and after treatment when intragroup values ere compared (PT+DG: *p* < 0.001; DG: *p* < 0.001; PTG: *p* < 0.001) No significant differences between groups in terms of VAS scores before and after treatment. Authors’ conclusions: “Pain sensation decreased in all groups and the quality of life of the patients increased after treatment in PTG. Intermittent diet may be an alternative option for the treatment of chronic pain.”

Abbreviations alphabetically ordered: ↑ = Increased; ↓ = Reduced; +: positively; -: negatively; BDI: Beck Depression Inventory; BI: Barthel Index; BMI: Body Mass Index; CFS: Chronic Fatigue Syndrome; CLBP: Chronic Low Back Pain; CPS: Central Pain Sensitivity; Ctrl: Control; DG: diet group; EA: Electroacupuncture; FIQ: Fibromyalgia Impact Questionnaire; LANSS: Leeds Assessment of Neuropathic Symptoms and Signs pain scale; MC1: Modic changes type 1; ME: Myalgic Encephalomyelitis; MFI: Multidimensional Fatigue Inventory; MMSE: Mini-Mental State Examination; NRS: Numerical rating scale for pain; NHP: Nottingham Health Profile; OA: Osteoarthritis; POMS: Profile of Mood States; PTG: Physical therapy group; PT+DG: Physical therapy + diet group; QoL: quality of Life; RMQ: Roland Morris Questionnaire; RPM: revolutions per minute; SA: Sham acupuncture; SF-12: The standard 12-item Short-Form Health Survey; SF-36: The SF-36 Quality of Life Questionnaire; STAI: 40-item State-Trait Anxiety Inventory; VAS: visual analog scale; W: watts; WOMAC: Western Ontario and McMaster Universities Osteoarthritis Index.

**Table 2:** Methodological quality evaluation of the clinical trials using the PEDro scale in randomized trials.

Scale “Physiotherapy Evidence Database (PEDro)” to Analyze the Methodological Quality of Clinical Studies												
Authors	Specified Selection Criteria	Randomization	Allocation Was Concealed	Similar Groups to Start	Blinded Subjects	Blinded Therapists	Blinded Raters	Outcomes 85%	Treatment or Intention to Treat	Comparison between Groups	Point Measures Variability	Outcome
Roman et al., 2018 [12]	Yes	Yes	Yes	Yes	Yes	Yes	Yes	Yes	Yes	Yes	Yes	10
Wang et al., 2021 [27]	Yes	Yes *	No	Yes	No	No	No	Yes	Yes	Yes	Yes	7
Jensen et al., 2019 [24]	Yes	Yes	Yes	Yes	Yes	Yes	No	No	Yes	Yes	Yes	9
Kenis-Coskun et al., 2020 [4]	Yes	No	No	Yes	Yes	Yes	No	No	Yes	Yes	Yes	7
Torlak et al., 2022 [26]	Yes	Yes	Yes	Yes	Yes	No	No	Yes	Yes	Yes	Yes	9

Yes *: randomization between both groups of patients. There is a third non-randomized control group. Result on the PEDro scale: 9–10 (excellent), 6–8 (good), 4–5 (acceptable) and <4 (poor).

**Table 3:** Methodological index for non-randomized studies (MINORS) to assess the methodological quality and risk of bias of the included observational studies. Items are scored 0 (not reported), 1 (reported but inadequate) or 2 (reported and adequate), with the global ideal score being 16 for non-comparative studies and 24 for comparative studies.

Methodological Index for Non-Randomized Studies (MINORS)													
Authors	A Clearly Stated Aim	Inclusion of Consecutive Patients	Prospective Collection of Data	Endpoints Appropriate to the Aim of the Study	Unbiased Assessment of the Study Endpoint	Follow-Up Period Appropriate to the Aim of the Study	Loss to Follow Up Less than 5%	Prospective Calculation of the Study Size	An Adequate Control Group *	Contemporary Groups *	Baseline Equivalence of Groups *	Adequate Statistical Analyses *	Outcome
Shukla et al., 2015 [18]	2	2	1	2	0	1	2	1	2	2	1	1	17

The items are scored 0 (not reported), 1 (reported but inadequate) or 2 (reported and adequate). The global ideal score being 16 for non-comparative studies and 24 for comparative studies. * Additional criteria in case of comparative study.

### 3.2. Outcomes

#### 3.2.1. Association between Gut Microbiome and Pain

The pain was measured through three different scales. Shukla et al. (2015) [18], Jensen et al. (2019) [24] and Wang et al. (2021) [27] used the numerical rating scale (NRS). Wang also used the Western Ontario and McMaster Universities Osteoarthritis Index (WOMAC) in the subscale of pain [27]. Roman et al. (2018) [12], Kenis-Coskun et al. (2020) [4] and Torlak et al. (2022) [26] chose the visual analog scale (VAS).

Shukla et al. (2015) [18] showed significant differences at baseline between the myalgic encephalomyelitis/chronic fatigue syndrome (ME/CFS) group and the control group in the relative abundance of the phylum Actinobacteria (higher in controls). It should be noted that ME/CFS patients experienced more pain (NRS mean: 6.8) than the control group (2.5).

Roman et al. (2018) [12] indicated a reduction in pain in the probiotic group compared to the placebo group but not statistically significant. Although they did not characterize the gut microbiome, this reduction is likely to be mediated by its modulation, as discussed by the authors (and suggested by the literature on the same probiotic strains used).

Jensen et al. (2019) [24] and Kenis-Coskun et al. (2020) [4] found improvement in pain after treatment; however, the differences were not significant. In particular, Jensen [24] did not show any differences between the control and active group in four out of five items: disability, back+leg pain, patient-reported global effect and number of the patients with minimal disability at 1 year. Nevertheless, authors highlighted that back pain decreased more in the active group than in the control group. Kenis-Coskum et al. [4] showed a decrease in the VAS median from 7/10 before treatment to 3/30 after treatment [4].

Wang et al. (2021) [27] mentioned a statistical difference between cases and controls at week 8 in NRS and WOMAC pain (i.e., lower values for cases). They also showed an overall reduction in pain during follow-up through week 26. The authors correlated this decrease with increased proportions of typically health-associated taxa, such as Faecalibacterium, Roseburia and Agathobacter (for WOMAC pain only). On the other hand, Streptococcus and Enterococcus, known pathobionts, were positively correlated with NRS and WOMAC pain.

Finally, Torlak et al. (2022) [26] found significant differences in VAS scores in intragroup comparing before and after treatment, but, in general terms before and after the treatment, there were not significant differences. As with Kenis-Coskum et al. [4], authors express the VAS in cm being the most important data for the VAS in the diet and physical therapy group before treatment 7.45 ± 0.44 and after treatment 4.7 ± 0.42 (<0.001).

#### 3.2.2. Association between the Gut Microbiome and Quality of Life

Roman et al. (2018) [12] and Wang et al. (2021) [27] measured quality of life with the SF-36 questionnaire. In particular, Wang et al. (2021) [27] used the abbreviated form, SF-12, which yields physical and mental health. According to their findings, both items improved during the follow-up. As for the gut microbiome, Blautia and Anaerostipes were positively correlated with SF-12, in the physiological and psychological scores. On the other hand, Roman et al. (2018) [12] showed better results in the SF-36 test for both the probiotic and placebo groups. No data on quality of life were provided by Shulka et al. (2015) [18].

Kenis-Coskun et al. (2020) [4] measured the quality of life through the Nottingham Health Profile (NHP) scale. This scale showed enhancements in five out of six items: energy, pain, emotional, sleep and physical being, with social life the only item where there were no improvements.

Torlak et al. (2022) [26] used the Barthel Index (BI) as a measure of quality of life using basic daily life activities. The disability in diet+physical therapy group and physical therapy group decreased significantly before and after treatment but not in the diet group.

#### 3.2.3. Association between the Gut Microbiome and the Exercise

There were several changes in the gut microbiome in all study participants after a maximal exercise challenge (Shukla et al., 2015 [18]). In the ME/CFS group, six out of the nine major taxa (mainly Clostridium cluster IV, clostridia, bacilli, Firmicutes and Actinobacteria) increased in stool from baseline at 72 h post-exercise compared with only two (Bacteroidetes and unclassified general) in the control group. The authors consistently observed rapid changes (i.e., increases) in Firmicutes levels in blood samples 15 min after maximal exercise, a phenomenon that might be more evident in patients than controls.

## 4. Discussion

The purpose of this systematic review was to summarize available evidence from clinical trials, linking lifestyle intervention and gut microbiome in CWP patients, a field still underexplored despite its promising potential. Although no filter was applied by the year of publication, only six trials had the minimum requirements to be included. Their results hypothesized favorable changes in gut microbiome composition due to lifestyle intervention (exercise vs. electroacupuncture vs. probiotics vs. vitamin D vs. diet changes). Overall, these changes were associated with improved pain experience and quality of life. Despite the different levels of taxonomic resolution among studies, microbiome variations mainly involved an increase in beneficial taxa, such as those producing short-chain fatty acids (mainly acetate, propionate and butyrate), which are microbial metabolites with a key multifactorial role in host physiology [28]. Despite the literature reporting some conflicting data [28,29], it is known that short-chain fatty acids have a general anti-inflammatory effect as well as immunoregulatory and neuromodulatory effects, acting at different levels along the gut–brain axis, thus potentially contributing to pain relief [30].

Regarding specific lifestyle interventions, a wide range of literature supports the therapeutic effects of exercise on pain intensity in CWP [31,32]. Recent bibliography also claims that exercise could modify the gut microbiome, with compositional and functional changes likely related to training variables, such as type, load, intensity, frequency, etc. [31,33,34]. In this regard, it should be noted that Shukla et al. (2015) [18] found evidence of bacterial translocation into the bloodstream after a maximal exercise challenge, thus suggesting that too intense exercise may not be entirely favorable.

On the other hand, probiotics are well-known microbiome manipulation tools with a long history of use [25]. However, both Roman et al. (2018) [12] and Jensen et al. (2019) [24] did not profile the gut microbiome and observed improvements even in the placebo group.

Treatment with vitamin D could be a suitable ally in the fight against CWP. Nevertheless, clinicians should take care regarding the quantity of vitamin D, as other authors reported that high levels of vitamin D could increase the pro-inflammatory mediators and, consequentially, increase the levels of pain [35].

Finally, it is hypothesized that electroacupuncture could slightly modify pain in knee OA because of its relationship with the inflammatory effects, reducing the quantity of Streptococcus. However, this association is still not clear enough [27]. Other authors also suggest the same hypothesis, however, manipulating directly the microbiome for modifying the knee OA pain [36].

CWP patients suffer from malfunctioning sensory processing in the CNS [37]. This may lead to deficiencies in the pain processing chain, such as descending pain-inhibitory mechanisms or temporal summation (TS) [38]. Not only functional changes, but the literature also explains structural neuroplastic changes related to this sensibilization [39]. Those changes could be promoted by the alterations in the gut microbiome, increasing the inflammatory response and chronification of the illness [5,36,40]. Therefore, there is a necessity of thinking about interventions that make an impact on the musculoskeletal, the microbiome and, as consequence, in the CNS [6,40,41].

It is complicated to draw firm conclusions about changes in pain sensitivity and gut microbiome solely due to the interventions mentioned before. For example, diet is a major microbiome-associated cofounding factor that, in many trials, has not been taken into account [34]. Additional research is therefore required to determine the best intervention to change microbiome composition and functionality towards a eubiotic profile capable of counteracting (i.e., decreasing) CWP. Such research should also provide insights into the underlying mechanisms, to be possibly validated in animal models.

It is important to highlight that despite all the interventions that have an impact in the microbiome, only three studies carried out a specific measurement of it. This fact leads us to explain that, in those where there is no analysis of the microbiome, its association is a hypothesis that will have to be contrasted with more scientific studies in this field.

This review has several limitations. One of them is the differences between protocols in order to measure the impact of each intervention on the microbiome. Although all the patients suffer from CWP, they were diagnosed with diverse illnesses. Appreciating those differences as well as the differences in the methodologies make this article out from the definition of a meta-analysis.

## 5. Conclusions

Lifestyle is one of the major variation drivers of the gut microbiome. Given the latter’s role in human pathophysiology, lifestyle interventions could result in microbiome-related improvements in pain and quality of life in several chronic widespread diseases.

## Data Availability

The data presented in this study are available on request from the corresponding authors.
